# The Impact of Non-Brazilian Contribution on the Publishing Performance of Psychology Journals in Brazil

**DOI:** 10.3389/fpsyg.2018.01934

**Published:** 2018-10-15

**Authors:** Chris Fradkin

**Affiliations:** ^1^Departamento de Psicologia, Pontifícia Universidade Católica do Rio de Janeiro, Rio de Janeiro, Brazil; ^2^Psychological Sciences, University of California, Merced, Merced, CA, United States

**Keywords:** internationalization, scientific journals, psychology, Brazil, bibliometric analysis

## Abstract

There is considerable variability in publishing performance among psychology journals in Brazil. However, research as to why is very scarce. This study empirically examined the relationship between non-Brazilian contribution and publishing performance, among these journals. A total of 746 articles from the top-18 psychology journals in Brazil were coded for study type, international collaboration, and non-Brazilian contribution. Analyses revealed that publishing performance was associated with the following: (i) international collaboration and (ii) non-Brazilian contribution. Collaboration with, and contribution from, English-speaking authors was more prevalent among the higher performing journals; while contribution from non-Brazilian Ibero-American authors was more prevalent among the lower performing journals. These findings suggest that publishing performance for psychology journals in Brazil may be strongly tied to non-Brazilian contribution. Implications may be relevant to journal publishers and editors, as well as arbiters of scientific policy.

## Introduction

There has been much written about “lost science” (Packer, 2001; Meneghini and Packer, 2007; Packer and Meneghini, 2007; Montgomery, 2013; Fradkin, 2017a), a term coined by Gibbs (1995) that refers to “the unaccessed scientific output of the ‘emerging’ or ‘developing’ nations (EDNs)” (Fradkin, 2015, p. 100). Among the EDNs, the BRICS nations, of Brazil, Russia, India, China, and South Africa, are a commonly referred to group that makes up 43% of the world’s population and 25% of its gross domestic product (Wilson and Purushothaman, 2003; Raghuramapatruni, 2015). Unfortunately, much of the scientific output from this group “remains inaccessible or ‘lost’ to many scholars in the English-speaking world” (Fradkin, 2015, p. 100), due to the fact that it is not published in English (Gibbs, 1995). In addition, the quality of these journals is typically lower than their lingua franca counterparts, due in large part to their less stable business model (Meneghini, 2012). Nonetheless, there is variability among these journals in their publishing performance. We will examine this variability among journals in the BRICS nation of Brazil. We will focus on publications in the field of psychology, an area which has experienced a boon in recent years (Hutz et al., 2004; Gamba et al., 2015).

For decades, Brazilian science has endured criticism from elitists in lingua franca quarters (Gibbs, 1995). In 2015, librarian Jeffrey Beall described these journals as residing on open-access platforms which “are more like publication favelas”^1^ (Beall, 2015). Nonetheless, over the last decade, a tiering of psychology journals in Brazil has come to be, with two of the top journals in the field now appearing on the global stage. These journals, *Psicologia: Reflexão e Crítica* and *Psychology & Neuroscience*, are now in partnerships with Springer and the American Psychological Association, both top-tiered international publishing houses. Through these partnerships, these journals now publish to a global audience. These international affiliations give these journals an advantage, when compared to their domestic counterparts. In short, they have: a wider dissemination through expanded databases; a higher level of translators and support team; and a higher prevalence of international submissions (Landeira-Fernandez et al., 2015; Remor, 2016). Because of these advantages, these two journals now perform at a higher level than their domestic counterparts. From this vantage, these two journals unwittingly provide an ideal for their sister journals to aspire to.

A recent study (Fradkin, 2017b) examined differences between these two internationally published journals and their lower tiered domestic counterparts. The findings indicated that these top two journals had a higher prevalence of lead authors from lingua franca/English-speaking countries than their lower tiered domestic counterparts (*U* = 0.00, *p* = 0.003). The findings also indicated that these journals had a higher prevalence of editorial board members from lingua franca/English-speaking countries (*U* = 1.50, *p* = 0.041). These findings provoked discussion in the field (see Medeiros, 2017; Nassi-Calò, 2017). There were queries about international collaboration. There were queries about non-Brazilian contribution.

While one might think this information is available online; after searching, one discovers it is not. There are exceptions though. There is the journal *Psychology & Neuroscience*, which ran an editorial in 2014 (Mograbi, 2014) that provided an international breakdown of reviewers and submissions. There is also the journal *Paidéia (Ribeirão Preto)*, which has run an international breakdown of ad hoc consultants and submissions since 2008 (Santos et al., 2008; Santos and de Oliveira, 2009; Santos, 2010, 2011, 2012, 2013, 2014, 2015, 2016, 2017). These withstanding, there has been no comprehensive tally of international collaboration or non-Brazilian contribution, among the psychology journals of Brazil. This absence is surprising, because these variables are linked inextricably to publishing performance (Smith et al., 2014; Bordons et al., 2015; Fradkin, 2015; Gamba et al., 2015).

With regard to international contribution, Rogério Meneghini, of the Scientific Electronic Library Online (SciELO), sees this variable as critical to high performing journals (Meneghini, 2013). In an examination of journal content from England, Netherlands, and Switzerland, Meneghini found that 85% was international. By contrast, in an examination of content from Brazil, China, and India, Meneghini found that only 20% was international (Meneghini, 2013). These findings suggest that international contribution is more prevalent among developed nation journals – those journals with the higher impact factors. And higher impact factors are synonymous in most circles with higher publishing performance (Packer, 2014; Bornmann et al., 2015; Fradkin, 2017a).

Regarding international collaboration, the findings are the same. In an examination of the most heavily cited Brazilian articles, SciELO’s Packer and Meneghini (2006) found that 84% were international collaborations, in contrast to 16% written solely by Brazilians. Of these articles, most of them were products of multinational collaboration (i.e., an author from Brazil and authors from at least two other countries). This relationship between international collaboration and strong publishing performance has also been documented in the United Kingdom (Adams et al., 2007), Asia (Arunachalam and Doss, 2000), Spain (Bordons et al., 2015), and Norway (Aksnes, 2003).

In sum, these studies indicate a positive relationship between: (a) international contribution and publishing performance (Meneghini, 2013; Packer, 2014; Bornmann et al., 2015), and (b) international collaboration and publishing performance (Arunachalam and Doss, 2000; Aksnes, 2003; Packer and Meneghini, 2006; Adams et al., 2007; Bordons et al., 2015). While we acknowledge that several studies have focused on these variables within the purview of Brazilian science broadly (e.g., Packer and Meneghini, 2006), there have been no studies that we know of that have examined these variables specifically among psychology journals in Brazil.

Therefore, this study aimed to examine the relationship between international contribution and collaboration and journal publishing performance among psychology journals in Brazil. Our hypotheses were based on the weight of findings from past studies. With regard to study type, we hypothesized that, in relation to their lower tiered domestic counterparts, the internationally published psychology journals in Brazil would have: (H1) a higher prevalence of international collaboration, (H2) a higher prevalence of non-Brazilian authored contributions, and (H3) a lower prevalence of non-collaborative Brazilian contributions. In the area of international collaboration, we hypothesized that the internationally published psychology journals in Brazil would have: (H4) a lower prevalence of Ibero-American collaborations and (H5) a higher prevalence of lingua franca collaborations. And finally, in the area of non-Brazilian contribution, we hypothesized that the internationally published psychology journals in Brazil would have: (H6) a lower prevalence of non-Brazilian Ibero-American contributions and (H7) a higher prevalence of non-Brazilian lingua franca contributions.

## Materials and Methods

A bibliometric analysis was conducted on the top-ranked psychology journals of Brazil, from the year 2016. The analysis assessed the relationship between international contribution and collaboration and the publishing performance of these journals.

### Sample

SCImago (SCImago, 2017) provides a ranking of scientific journals based on the number of citations received by a journal and the importance or prestige of the journals where such citations came from. Of the 1,063 worldwide psychology journals listed in the 2015 SCImago Journal Ranking (SJR), 18 (1.7%) were included from Brazil. These journals supplied the articles that comprised this study’s sample. From the output of these journals for the year 2016 (814 articles), 746 research articles were included in the study. Excluded were as follows: editorials, book reviews, interviews, corrections, letters, obituaries, and re-publications of historical works.

### Variables of Interest

*International collaboration* was recorded dichotomously (yes/no) for each article of each issue of each journal for the year. Articles co-authored by a scholar affiliated with a Brazilian institution and at least one scholar affiliated with a non-Brazilian institution met the criteria for international collaboration.

*Brazilian authored articles* refers to articles authored solely by scholars affiliated with Brazilian institutions. This classification was recorded dichotomously (yes/no) for each article of each issue of each journal for the year.

*Non-Brazilian authored articles* refers to articles authored solely by scholars affiliated with non-Brazilian institutions. Non-Brazilian authored status was recorded dichotomously (yes/no) for each article of each issue of each journal for the year. Prevalence of non-Brazilian authored articles was calculated separately for the two comparison groups (see publication house) and for the sample as a whole: *n* articles_NON-BRAZIL-AUTH_ ÷ *n* articles_TOTAL_.

*Region of article* was determined by country of authorship. International collaborations and non-Brazilian contributions were coded: (1) Ibero-American, for articles by authors based in Spanish- and Non-Brazilian Portuguese-speaking countries (South and Central America, Mexico, Caribbean, Spain, Portugal, Angola, Mozambique); (2) Lingua Franca, for articles by authors based in native English-speaking countries (United States, United Kingdom, Canada, South Africa, Australia, New Zealand); and (3) others, for articles that did not meet the previous criteria. Articles received more than one code, if authors were based in different regions.

### Grouping Variable

*Publication house* defined the structure through which the journal disseminates its work. This information was gathered from each journal’s web site and recorded dichotomously (international publication house/no international publication house). This variable defined our two groups for comparison.

### Procedure

#### Data Collection

Research articles (e.g., research reports, reviews, and theoretical articles) were included in the study. Non-research articles (e.g., editorials, book reviews, interviews, corrections, letters, obituaries, and re-publication of historical works) were excluded. From a starting pool of 814 articles, 68 non-research articles were excluded resulting in 746 research articles for the sample. Articles were first coded for study type (international collaboration, Brazilian authored, and non-Brazilian authored). They were then coded for region (Ibero-American, lingua franca, and others), with articles receiving more than one code, if authors came from different regions. Information on the journals’ publication house was gathered from the journals’ web site. As two of the journals were published through international publishing houses, we used the variable publication house (international or Brazilian) to differentiate the journals of higher (*n* = 2) from those of lower (*n* = 16) performance status.

#### Data Analysis

Analyses were performed using SPSS version 16.0. First, frequencies of study type (international collaboration, Brazilian authored articles, and non-Brazilian authored articles) were tallied and converted to percentages of the sample’s total articles (e.g., international collaboration = *n* articles_INT-COLLAB_ ÷ *n* articles_TOTAL_). Frequencies of these variables were then tallied within the two comparison groups (Brazilian publication house/international publication house) and converted to percentages as before. Next, frequencies of region (Ibero-American, lingua franca, and others) were tallied and converted to percentages of the sample’s articles of international collaboration (e.g., Ibero-American = *n* articles_IBERO-AMERICAN-NON-BRAZILIAN_ ÷ *n* articles_TOTAL-INT-COLLAB_). Frequencies of these variables were then tallied within the two comparison groups and converted to percentages as before. Next, frequencies of region were tallied and converted to percentages of the sample’s articles of non-Brazilian contribution (e.g., Ibero-American = *n* articles_IBERO-AMERICAN-NON-BRAZILIAN_ ÷ *n* articles_TOTAL-NON-BRAZILIAN_). Frequencies of these variables were then tallied within the two comparison groups and converted to percentages. Chi-square tests were then conducted on each variable to determine significant differences between comparison groups. In one case, which had an observed cell count of zero, a Fisher’s exact test was performed in lieu of Chi-square. As all variables were in dichotomous format, testing was conducted within 2 × 2 contingency tables, with alpha set at 0.05.

## Results

### Descriptive Statistics

The 18 journals of **Table 1** supplied the sample’s articles. Of these journals, three publish articles exclusively in English; three in Portuguese; and the remaining in a hybrid format. Nine of the journals are indexed by the SciELO database, which attests to their presence in the Latin American and Caribbean markets. Of the journals not indexed by SciELO, it should be noted that *Psychology & Neuroscience* withdrew from SciELO upon establishing a partnership with an international publishing house. Also shown is the Qualis indicator. Qualis is a ranking system administered by Coordenação de Aperfeiçoamento de Pessoal de Nível Superior (CAPES)^2^ for the evaluation of Brazil’s scientific journals. Among the journals of our sample, all but one received this system’s first or second highest rating (A1 or A2, respectively). And critical to the study is publication house assignment, which places the two journals published by international publishing houses in the top third of the sample.

**Table 1:** Descriptive statistics of the top Brazilian psychology journals.

Rank	Journal	Est.	Impact Factor	Text Lang.	SciELO Inclusion	Qualis Rating	Publication House
1	*Psicologia: Reflexão e Crítica*	1988	0.32	Eng.	Yes	A1	Int’l
2	*Psicologia e Sociedade*	1986	0.09	Hyb.	Yes	A2	Brazil
3	*Psicologia Escolar e Educacional*	1996	0.18	Hyb.	Yes	A2	Brazil
4	*Revista Latinoamericana de Psicopatologia Fundamental*	1998	0.08	Hyb.	Yes	A2	Brazil
5	*Psicologia: Teoria e Pesquisa*	1985	0.31	Hyb.	Yes	A1	Brazil
6	*Psychology & Neuroscience*	2008	0.27	Eng.	No^b^	A2	Int’l
7	*Revista Brasileira de Orientação Profissional*	2000	0.04	Hyb.	No	A2	Brazil
8	*Paidéia (Ribeirão Preto)*	1991	0.24	Eng.	Yes	A1	Brazil
9	*Psicologia em Estudo*	1996	0.18	Hyb.	No^c^	A1	Brazil
10	*Estudos de Psicologia (Natal)*	1996	0.16	Hyb.	Yes	A1	Brazil
11	*Psicologia USP*	1990	0.09	Hyb.	Yes	A2	Brazil
12	*Psicologia Clínica*	1989	0.02	Hyb.	No^d^	A2	Brazil
13	*Temas em Psicologia*	1993	0.13	Hyb.	No	A2	Brazil
14	*Arquivos Brasileiros de Psicologia*	1949	0.08	Por.	No	A2	Brazil
15	*Revista Brasileira de Neurologia e Psiquiatria*	1997	0.00	Hyb.	No	B4^e^	Brazil
16	*Ágora: Estudos em Teoria Psicanalítica*	1998	0.00	Hyb.	Yes	A2	Brazil
17	*Tempo Psicanalítico*	1978	0.02	Por.	No	A2	Brazil
18	*Revista da Abordagem Gestáltica*	1995	0.02	Por.	No	A2	Brazil

*Rankings by SCImago (for 2015); Est., established; Impact Factor, 2015 citations of articles published 2013–14; Text Lang., article text body language; Eng., 100% text body English; Hyb., hybrid/assorted text body languages; Por., 100% text body Portuguese; Int’l, international.*

*^a^Qualis Classificação de Periódicos 2015 Psicologia rating (except when noted).*

*^b^Indexed in SciELO 2008–2014; withdrawn from SciELO upon partnership with APA in 2015.*

*^c^Indexed in SciELO 2000–2014; indexing interrupted Sept., 2015.*

*^d^Indexed in SciELO 2005–2015; indexing interrupted Sept., 2015.*

*^e^Qualis Classificação de Periódicos 2015 Medicina I rating.*

### Association Between Performance and Study Type (Hypotheses 1–3)

**Table 2** presents a breakdown of the study types by journal, while **Table 3** presents the data across groups. Hypothesis 1 predicted that there would be a higher prevalence of international collaboration among the internationally published journals than among their lower tiered domestic counterparts. A chi-square test revealed that the prevalence of international collaboration was higher among the internationally published journals (*M* = 13.4%) than among their lower tiered domestic counterparts (*M* = 5.3%), χ^2^(1) = 8.37, *p* = 0.004. In the presence of a significant main effect, Hypothesis 1 was supported.

**Table 2:** Top Brazilian psychology journals: study types.

Journal	Total	Int’l Collab.		Braz. Auth.		Non-Braz. Auth.	
	Docs.	*n*	%	*n*	%	*n*	%
*Psicologia: Reflexão e Crítica*	47	7	14.9	29	61.7	11	23.4
*Psicologia e Sociedade*	60	3	5.0	50	83.3	7	11.7
*Psicologia Escolar e Educacional*	62	3	4.8	49	79.0	10	16.1
*Revista Latinoamericana de Psicopatologia Fundamental*	36	1	2.8	30	83.3	5	13.9
*Psicologia: Teoria e Pesquisa*	82	10	12.2	64	78.0	8	9.8
*Psychology & Neuroscience*	35	4	11.4	16	45.7	15	42.9
*Revista Brasileira de Orientação Profissional*	10	1	10.0	6	60.0	3	30.0
*Paidéia (Ribeirão Preto)*	39	0	0.0	31	79.5	8	20.5
*Psicologia em Estudo*	61	5	8.2	55	90.2	1	1.6
*Estudos de Psicologia (Natal)*	44	2	4.5	41	93.2	1	2.3
*Psicologia USP*	56	4	7.1	44	78.6	8	14.3
*Psicologia Clínica*	26	1	3.8	19	73.1	6	23.1
*Temas em Psicologia*	80	3	3.8	70	87.5	7	8.8
*Arquivos Brasileiros de Psicologia*	20	1	5.0	18	90.0	1	5.0
*Revista Brasileira de Neurologia e Psiquiatria*	16	1	6.3	14	87.5	1	6.3
*Ágora: Estudos em Teoria Psicanalítica*	35	0	0.0	28	80.0	7	20.0
*Tempo Psicanalítico*	13	0	0.0	13	100.0	0	0.0
*Revista da Abordagem Gestáltica*	24	0	0.0	24	100.0	0	0.0
Total	746	46	6.2	601	80.6	99	13.3

*Int’l Collab., international collaboration; Braz. Auth., authored solely by Brazilian(s); Non-Braz. Auth., authored by non-Brazilian(s). Total % may not match sums because of rounding.*

**Table 3:** Top Brazilian psychology journals: articles by study type and journal.

Study type	Total sample		Lower tiered		Higher tiered	
	*n*	%	*n*	%	*n*	%
Int’l collaboration	46	6.2	35	5.3	11	13.4
Brazilian authored	601	80.6	556	83.7	45	54.9
Non-Brazilian authored	99	13.3	73	11.0	26	31.7
Total	746	100.0	664	100.0	82	100.0

*Lower tiered includes articles from journals affiliated with Brazilian publishing houses (n = 16); higher tiered includes articles from journals affiliated with international publishing houses (n = 2). Int’l, international. Total % may not match sums because of rounding.*

Hypothesis 2 predicted that there would be a higher prevalence of non-Brazilian authored contributions among the internationally published journals than among their lower tiered domestic counterparts. A chi-square test revealed that the prevalence of non-Brazilian authored contributions was higher among the internationally published journals (*M* = 31.7%) than among their lower tiered domestic counterparts (*M* = 11.0%), χ^2^(1) = 27.21, *p* < 0.001. In the presence of a significant main effect, Hypothesis 2 was supported.

Hypothesis 3 predicted that there would be a lower prevalence of non-collaborative Brazilian contributions among the internationally published journals than among their lower tiered domestic counterparts. A chi-square test revealed that the prevalence of non-collaborative Brazilian contributions was lower among the internationally published journals (*M* = 54.9%) than among their lower tiered domestic counterparts (*M* = 83.7%), χ^2^(1) = 38.81, *p* < 0.001. In the presence of a significant main effect, Hypothesis 3 was supported.

### Association Between Performance and International Collaboration (Hypotheses 4–5)

**Table 4** presents collaboration across groups. Hypothesis 4 predicted that there would be a lower prevalence of Ibero-American collaboration among the internationally published journals than among their lower tiered domestic counterparts. A chi-square test revealed that the prevalence of Ibero-American collaboration was not different between the internationally published journals (*M* = 27.3%) and their lower tiered domestic counterparts (*M* = 54.3%), χ^2^(1) = 2.45, *p* = 0.118. In the absence of a significant main effect, Hypothesis 4 was not supported.

**Table 4:** Top Brazilian psychology journals: international collaboration by region and journal.

Region	Total sample		Lower tiered		Higher tiered	
	*n*	%	*n*	%	*n*	%
Ibero-American	22	47.8	19	54.3	3	27.3
Lingua Franca	18	39.1	10	28.6	8	72.7
Others	10	21.7	7	20.0	3	27.3
Total	50	108.7	36	102.9	14	127.3

*Lower tiered includes articles from journals affiliated with Brazilian publishing houses (n = 16); higher tiered includes articles from journals affiliated with international publishing houses (n = 2); Ibero-American includes Spanish- and Portuguese-speaking countries (South and Central America, Mexico, Caribbean, Spain, Portugal, Angola, and Mozambique); Lingua Franca includes native English-speaking countries (United States, United Kingdom, Canada, and South Africa). The total sums exceed those of **Table 2** because of multinational collaborations. Total % may not match sums because of rounding.*

Hypothesis 5 predicted that there would be a higher prevalence of lingua franca collaboration among the internationally published journals than among their lower tiered domestic counterparts. A chi-square test revealed that the prevalence of lingua franca collaboration was higher among the internationally published journals (*M* = 72.7%) than among their lower tiered domestic counterparts (*M* = 28.6%), χ^2^(1) = 6.85, *p* = 0.009. In the presence of a significant main effect, Hypothesis 5 was supported.

### Association Between Performance and Non-Brazilian Contributions (Hypotheses 6–7)

**Table 5** presents non-Brazilian contributions across groups. Hypothesis 6 predicted that there would be a lower prevalence of non-Brazilian Ibero-American contributions among the internationally published journals than among their lower tiered domestic counterparts. A chi-square test revealed that the prevalence of non-Brazilian Ibero-American contributions was lower among the internationally published journals (*M* = 53.8%) than among their lower tiered domestic counterparts (*M* = 82.2%), χ^2^(1) = 8.16, *p* = 0.004. In the presence of a significant main effect, Hypothesis 6 was supported.

**Table 5:** Top Brazilian psychology journals: non-Brazilian contributions by region and journal.

Region	Total sample		Lower tiered		Higher tiered	
	*n*	%	*n*	%	*n*	%
Ibero-American	74	74.7	60	82.2	14	53.8
Lingua Franca	12	12.1	0	0.0	12	46.2
Others	22	22.2	18	24.7	4	15.4
Total	108	109.1	78	106.8	30	115.4

*Lower tiered includes articles from journals affiliated with Brazilian publishing houses (n = 16); higher tiered includes articles from journals affiliated with international publishing houses (n = 2); Ibero-American includes Spanish- and Portuguese-speaking countries (South and Central America, Mexico, Caribbean, Spain, Portugal, Angola, Mozambique, etc.); Lingua Franca includes native English-speaking countries (United States, United Kingdom, Canada, South Africa, Australia, New Zealand, etc.). The total sums exceed those of **Table 2** because of multinational collaborations. Total % may not match sums because of rounding.*

Hypothesis 7 predicted that there would be a higher prevalence of non-Brazilian lingua franca contributions among the internationally published journals than among their lower tiered domestic counterparts. A Fisher’s exact test revealed that the prevalence of non-Brazilian lingua franca contributions was higher among the internationally published journals (*M* = 46.2%) than among their lower tiered domestic counterparts (*M* = 0.0%), *p* < 0.001. In the presence of a significant main effect, Hypothesis 7 was supported.

## Discussion

This study is the first empirical examination of international contribution and collaboration and its relationship with publishing performance among psychology journals in Brazil. Consistent with Hypothesis 1, as well as recent research (Adams et al., 2007; Bordons et al., 2015), is the finding of a higher prevalence of international collaboration among the internationally published journals in the sample. Consistent with Hypothesis 2, as well as recent research (Packer, 2014; Bornmann et al., 2015), is the finding of a higher prevalence of non-Brazilian authored contributions among the internationally published journals, in relation to their domestic counterparts. Consistent with Hypothesis 3, as well as recent research (Meneghini, 2013; Packer, 2014), is the finding of a lower prevalence of non-collaborative Brazilian contributions among the internationally published journals in the sample. These findings indicate that non-Brazilian input, be it collaborative or non-Brazilian authored, is associated with higher journal/publishing performance. These findings are also consistent with a recent study (Fradkin, 2017b) that found a higher prevalence of lead authors from lingua franca/English-speaking countries among those journals of higher internationalization. From these parallel trajectories, the critical thinker might infer that internationalization and performance are related. If so, this thinking would be consistent with the government’s evaluation standards for its graduate programs (Bianco et al., 2015; Gomes and Fradkin, 2015) and its scientific journals (Guzzo et al., 2015; Neto et al., 2016).

Within international collaboration, our findings yielded mixed results. Inconsistent with Hypothesis 4, as well as recent research (Paiva et al., 2017), is the finding that there is not a lower prevalence of Ibero-American collaboration among the internationally published journals in our sample. In hindsight, this might be expected, as the language of Brazil is most compatible with dialects from Ibero-American countries. Consistent with Hypothesis 5, as well as recent research (Bordons et al., 2015), is the finding of a higher prevalence of lingua franca collaboration among the internationally published journals. This finding of a higher lingua franca presence is consistent with the previously mentioned study (Fradkin, 2017b) that found a higher prevalence of lead authors from lingua franca/English-speaking countries among those journals of higher internationalization. Taken with Finding 4, one might infer it is the *presence* of lingua franca input, rather than the *absence* of Ibero-American input, that serves as fuel for the higher performing journals.

Within non-Brazilian contributions, our findings brought hypothesized results. Consistent with Hypothesis 6, as well as recent research (Loughborough et al., 2016), is the finding of a lower prevalence of non-Brazilian Ibero-American contributions among the internationally published journals in the sample. And consistent with Hypothesis 7, as well as recent research (Loughborough et al., 2016; González-Álvarez and Cervera-Crespo, 2017), is the finding of a higher prevalence of non-Brazilian lingua franca contributions among the internationally published journals in the sample. These last two findings highlight two points that serve as bookends: (1) there is a *positive* relationship between the publishing of material from non-Brazilian lingua franca countries and higher journal/publishing performance; and (2) there is a *negative* relationship between the publishing of material from non-Brazilian Ibero-American countries and higher journal/publishing performance. These last two findings reaffirm the value that non-Brazilian lingua franca studies have for psychology journals in Brazil.

As a whole, these findings suggest that the performance of psychology journals in Brazil is strongly linked to contributions from non-Brazilian quarters. When taken with the findings (Fradkin, 2017b) that the higher tiered psychology journals in Brazil have a higher prevalence of editorial bohese implications are powerfully compelling (see **Table 6**). In fact, they may serve as a blueprint for those editors and publishers who aspire to reach a global readership. For them, the message is: to better reach the global market, you must embrace non-Brazilian contribution. The findings of our study may also be of value to the arbiters of policy. For those involved with Qualis ranking, the message should ring clear: to better rank Brazilian journals, your criteria should include the value of non-Brazilian contribution.

**Table 6:** Top Brazilian psychology journals: contrasts across performance levels.

Lower tiered journals	Higher tiered journals
Brazilian publishing house	International publishing house
Hybrid-language format	English-language format
↓ International collaboration	↑ International collaboration
↓ Non-Brazilian contribution	↑ Non-Brazilian contribution
↑ Non-collaborative Brazilian contribution	↓ Non-collaborative Brazilian contribution
↓ Lingua franca collaboration	↑ Lingua franca collaboration
↑ Non-Brazilian Ibero-American contribution	↓ Non-Brazilian Ibero-American contribution
↓ Non-Brazilian lingua franca contribution	↑ Non-Brazilian lingua franca contribution
↓ Empirical studies^†^	↑ Empirical articles^†^
↓ Lingua franca lead-authored articles^†^	↑ Lingua franca lead-authored articles^†^
↓ Lingua franca editorial board members^†^	↑ Lingua franca editorial board members^†^

*Lower tiered journals, journals affiliated with Brazilian publishing houses (n = 16); higher tiered journals, journals affiliated with international publishing houses (n = 2); ↑, higher %; ↓, lower %.*

*^†^Based on findings from Fradkin, 2017b.*

For some, the notion of non-Brazilian contribution may be threatening. “They’re depriving our journals of our [Brazilian] content,” they may say. For those editors and publishers, it should be stressed that non-Brazilian contributions should *supplement* their now existing content (Fradkin, 2017b). In terms of collaboration, we are by no means suggesting that Brazilians should only work with lingua franca scholars. Rather, we are suggesting that Brazilians *supplement* their present pool, with non-Brazilians that speak fluent lingua franca. Programs are in place that promote such collaborations. The Fulbright Association (Fulbright Brasil, 2018), for one, offers grants for Brazilian scholars to develop their research in the United States. Additionally, CAPES offers funding for Brazilian scholars to pursue graduate and post-graduate studies overseas (Fundação CAPES, 2018).

These travel opportunities are key for Brazilian scholars, who must master English to build an international career (Meneghini and Packer, 2007). For them, their value as a scholar is gauged by their publication record in high impact factor, English-language journals (Lehmann et al., 2006; Mazloumian, 2012). Thus, their language presentation when submitting manuscripts must be competitive with native English-language scholars (Meneghini and Packer, 2007; Fradkin, 2017a). This brings us back to the notion of lost science. With the higher impact factor journals publishing in English, the acceptance rate for non-native English speaking scholars is much lower than for their lingua franca counterparts (Vasconcelos et al., 2007; Paiva et al., 2017). Likewise, the publishing performance for emerging nation journals is much lower than for their lingua franca counterparts (Tijssen et al., 2006; Packer, 2014; Fradkin, 2015, 2017b). This reality creates a less-than-even playing field for the dissemination of newly published science. Much science published in emerging nation mother tongue is unavailable in English for lingua franca readers. Conversely, English language science is available, but is only readable by emerging nation scholars who read English (Gibbs, 1995). This wall in both directions is the gist of lost science; and the solution is far from being present.

The recommendations, from our findings, are that a subset of emerging nation journals adopt the hallmarks of the higher performing journals. We define this subset as journals that: (1) are publishing content of a global (v. regional) nature; (2) have an administrative staff comfortable in English; and (3) have a natural affinity for lingua franca publication. Of the lower tiered journals in our sample, the one that fits these criteria most closely would be *Paidéia*, based on its publishing of articles exclusively in English (see **Table 1**). Among the remaining lower tiered journals, the ones that meet these criteria most weakly would be the three that publish articles exclusively in Portuguese. Thus, the recommendations that we offer would be intended for *Paidéia* and a portion of the hybrid-language journals in our sample (see **Table 1**).

These recommendations, however, give pause to several questions. Did the non-Brazilian authors submit their articles to the higher tiered Brazilian journals because of the journals’ higher quality and status? Or did the publication of submissions from non-Brazilian authors elevate the quality and status of the journal? The answer to these questions: “It depends.” In addition to directionality, a related issue would be the path the journal takes if it aspires to reach the global market. Should the focus of the journal be on upgrading its quality? Or should it be in attracting non-Brazilian authors to submit?

The answers to these questions are not easy to untangle. Through SciELO, Meneghini and Packer (2007) work to upgrade the quality of Brazilian science. We should remember, however, that among the journals in Brazil, not all of them were conceived as global instruments. For the most part, they were conceived within the confines of the university, as voices of the graduate departments (Féres-Carneiro et al., 2018). As such, they featured work from local scientists and scholars – work more Brazilian in its scope than international. Thus, the system that sustained them is different than the models of today’s global publishers (e.g., Elsevier, Sage, and Springer) that are structured as commercial enterprises (Hutz et al., 2004; Meneghini, 2012). Thus said, the recovery of lost science is not a simple undertaking, in spite of open access and today’s technology.

Among limitations in this study, the first would be materials acquisition. The assemblage of the articles began in January 2017, but by June, three journals in the sample had yet to publish their last 2016 issue^3^. For our findings, analyses were run with materials outstanding from these three; however, we have no reason to believe that the outstanding articles were significantly different from those included in the study. Of interest is the fact that two of these three journals publish articles exclusively in Portuguese. Another limitation would be in the coding of article type by institutional affiliation. We acknowledge that this would lead to articles by Brazilian nationals working at international institutions being coded “international” and not “Brazilian.” Nonetheless, with an author roster of our size, a non-institutional coding was not feasible. Another limitation would be the correlational nature of the study, from which causality cannot be inferred. And, as this study is a snapshot of only one year’s worth of issues, we should refrain from projecting outcomes for the future. But, despite these limitations, our findings serve a purpose that may ripple past the borders of Brazil.

## Conclusion

Opportunities exist for emerging nation science as they never have before. Collaborations can be brokered through research network sites (e.g., Academia, ResearchGate, and Mendeley); articles can be accessed on the Net. Through today’s technology, the emerging nation journal can disseminate its findings to the world. Our findings suggest that publishing performance for psychology journals in Brazil may be tied to non-Brazilian contribution. Having said that, we acknowledge that variability exists among the variety of emerging nation journals. Thus, the recommendations that we offer are primarily for those journals that have the content, staff, and lingua franca temperament, that lend themselves to global publication. For the publishers and editors of journals of this type, these recommendations are respectfully directed.

## Author Contributions

The author confirms being the sole contributor of this work and has approved it for publication.

## Conflict of Interest Statement

The author declares that the research was conducted in the absence of any commercial or financial relationships that could be construed as a potential conflict of interest.

## Footnotes

1 Favela is the Brazilian term for shantytown or slum.

2 CAPES is the agency affiliated with the Brazilian Ministry of Education that oversees graduate education programs.

3 Publication delay is problematic with Brazilian journals, overall. It also highlights the shortcomings of the nation’s publication model, which depends on academic (vs. commercial) funding for sustenance.

## References

[B1] Adams J. Gurney K. Marshall S. (2007). *Patterns of International Collaboration for the UK and Leading Partners.* Leeds: Evidence Ltd.

[B2] Aksnes D. W. (2003). Characteristics of highly cited papers. *Res. Eval.* 12 159–170. 10.3152/147154403781776645

[B3] Arunachalam S. Doss M. J. (2000). Mapping international collaboration in science in Asia through coauthorship analysis. *Curr. Sci.* 79 621–628.

[B4] Beall J. (2015). *Is SciELO a Publication Favela? Emerald City Journal.* Available at: https://www.emeraldcityjournal.com/2015/07/is-scielo-a-publication-favela/ [accessed July 23 2018].

[B5] Bianco A. C. L. Hutz C. S. Yamamoto M. E. (2015). Internationalization: towards new horizons. *Psicologia* 28(Suppl. 1), 49–56. 10.1590/1678-7153.2015284008

[B6] Bordons M. González-Albo B. Aparicio J. Moreno L. (2015). The influence of R&D intensity of countries on the impact of international collaborative research: evidence from Spain. *Scientometrics* 102 1385–1400. 10.1007/s11192-014-1491-4

[B7] Bornmann L. Wagner C. Leydesdorff L. (2015). BRICS countries and scientific excellence: a bibliometric analysis of most frequently cited papers. *J. Assoc. Inform. Sci. Technol.* 66 1507–1513. 10.1002/asi.23333

[B8] Féres-Carneiro T. Gomes W. B. Hutz C. S. Remor E. (2018). Editorial to 30th anniversary: historical links between the Psicologia: Reflexão e Crítica and the postgraduate psychology program at Universidade Federal do Rio Grande do Sul (Brazil). *Psicologia* 31:18 10.1186/s41155-018-0098-8 PMC6966914 32026083

[B9] Fradkin C. (2015). A summary evaluation of the top-five Brazilian psychology journals by native English-language scholars. *Psicologia* 28(Suppl. 1), 99–111. 10.1590/1678-7153.20152840014

[B10] Fradkin C. (2017a). Scientific publication for nonnative English speakers: a retrospective of a workshop in Brazil. *Sci. Commun.* 39 395–403. 10.1177/1075547017710244

[B11] Fradkin C. (2017b). The internationalization of psychology journals in Brazil: a bibliometric examination based on four indices. *Paidéia* 27 7–15. 10.1590/1982-43272766201702

[B12] Fulbright Brasil (2018). *Professor/Pesquisador Visitante nos EUA [Web page].* Available at: http://fulbright.org.br/edital/professorpesquisador-visitante/

[B13] Fundação CAPES (2018). *Ministério da Educação. Bolsas/Estudantes [Web page].* Available at: http://www.capes.gov.br/bolsas

[B14] Gamba E. C. Packer A. L. Meneghini R. (2015). Pathways to internationalize Brazilian journals of psychology. *Psicologia* 28(Suppl. 1), 66–71. 10.1590/1678-7153.20152840010

[B15] Gibbs W. W. (1995). Lost science in the Third World. *Sci. Am.* 273 92–99. 10.1038/scientificamerican0895-92

[B16] Gomes W. B. Fradkin C. (2015). Historical notes on psychology in Brazil: the creation, growth and sustenance of postgraduate education. *Psicologia* 28(Suppl. 1), 2–13. 10.1590/1678-7153.2015284002

[B17] González-Álvarez J. Cervera-Crespo T. (2017). Research production in high-impact journals of contemporary neuroscience: a gender analysis. *J. Informetr.* 11 232–243. 10.1016/j.joi.2016.12.007

[B18] Guzzo R. S. L. Linhares M. B. M. Teodoro M. L. M. Koller S. H. (2015). Perspectives and challenges regarding Brazilian policies for research and postgraduate studies in psychology. *Psicologia* 28(Suppl. 1), 34–39. 10.1590/1678-7153.2015284006

[B19] Hutz C. McCarthy S. Gomes W. (2004). “Psychology in Brazil: the road behind and the road ahead,” in *Handbook of International Psychology*, eds Stevens M. J. Wedding D. (New York, NY: Brunner-Routledge), 151–168.

[B20] Landeira-Fernandez J. Cruz A. Ventura D. F. (2015). Looking to the future: The American Psychological Association is the new publisher of psychology & neuroscience. *Psychol. Neurosci.* 5 1–2. 10.1037/pne0000012

[B21] Lehmann S. Jackson A. D. Lautrup B. E. (2006). Measures for measures. *Nature* 444:1003. 10.1038/4441003a 17183295

[B22] Loughborough W. Dale H. Wareham J. H. Youssef A. H. Rodrigues M. A. Rodrigues J. C. (2016). Characteristics and trends in publication of scientific papers presented at the European Congress of Radiology: a comparison between 2000 and 2010. *Insights Imaging* 7 755–762. 10.1007/s13244-016-0511-8 27484995 PMC5028340

[B23] Mazloumian A. (2012). Predicting scholars’ scientific impact. *PLoS One* 7:e49246. 10.1371/journal.pone.0049246 23185311 PMC3504022

[B24] Medeiros C. (2017). *Internacionalização para além dos artigos em inglês [Blog post].* Available at: https://www.blogs.unicamp.br/cienciaemrevista/2017/03/03/internacionalizacao/ [accessed February 12 2018].

[B25] Meneghini R. (2012). Emerging journals: the benefits of and challenges for publishing scientific journals in and by emerging countries. *EMBO Rep.* 13 106–108. 10.1038/embor.2011.252 22240975 PMC3271339

[B26] Meneghini R. (2013). Scielo, scientific electronic library online, a database of open access journals. *High. Learn. Res. Commun.* 3 3–7. 10.18870/hlrc.v3i3.153 27231797

[B27] Meneghini R. Packer A. L. (2007). Is there science beyond English? Initiatives to increase the quality and visibility of non-English publications might help to break down language barriers in scientific communication. *EMBO Rep.* 8 112–116. 10.1038/sj.embor.7400906 17268499 PMC1796769

[B28] Mograbi D. C. (2014). Psychology & Neuroscience indicators in 2013: evidence of growth and internationalization. *Psychol. Neurosci.* 7 61–63. 10.3922/j.psns.2014.022

[B29] Montgomery S. L. (2013). *Does Science Need a Global Language?: English and the Future of Research.* Chicago: University of Chicago Press 10.7208/chicago/9780226010045.001.0001

[B30] Nassi-Calò L. (2017). *Internacionalização como Indicador de Desempenho de Periódicos do Brasil: O caso da Psicologia [Blog post].* Available at: http://blog.scielo.org/blog/2017/03/14/internacionalizacao-como-indicador-de-desempenho-de-periodicos-do-brasil-o-caso-da-psicologia/ [accessed January 3 2018].

[B31] Neto S. C. Willinsky J. Alperin J. P. (2016). Measuring, rating, supporting, and strengthening open access scholarly publishing in brazil. *Educ. Policy Anal. Arch.* 24 1–25. 10.14507/epaa.24.2391

[B32] Packer A. L. (2001). “The SciELO Model for electronic publishing and measuring of usage and impact of Latin American and Caribbean scientific journals,” in *Proceedings of the Second ICSU/UNESCO International Conference on Electronic Publishing in Science*, (Paris: ICSU Press), 53–56.

[B33] Packer A. L. (2014). The emergence of journals of Brazil and scenarios for their future. *Educ. Pesqui.* 40 301–323.

[B34] Packer A. L. Meneghini R. (2006). Articles with authors affiliated to Brazilian institutions published from 1994 to 2003 with 100 or more citations: I-the weight of international collaboration and the role of the networks. *Anais Acad. Bras. Ciênc.* 78 841–853. 10.1590/S0001-37652006000400017 17143417

[B35] Packer A. L. Meneghini R. (2007). Learning to communicate science in developing countries. *Interciencia* 32:643.

[B36] Paiva C. E. Araujo R. L. Paiva B. S. R. de Pádua Souza C. Cárcano F. M. Costa M. M. (2017). What are the personal and professional characteristics that distinguish the researchers who publish in high-and low-impact journals? A multi-national web-based survey. *Ecancermedicalscience* 11:718. 10.3332/ecancer.2017.718 28194230 PMC5295845

[B37] Raghuramapatruni R. (2015). revealed comparative advantage and competitiveness: a study on BRICS. *Arabian J. Bus. Manage. Rev.* 5 1–7.

[B38] Remor E. (2016). A new era for the journal Psicologia: Reflexão e Crítica. *Psicologia* 29:1 10.1186/s41155-016-0011-2 PMC10575829 37831209

[B39] Santos M. A. D. (2010). Paidéia: relatório de gestão-2009. *Paidéia* 20 3–6. 10.1590/S0103-863X2010000100002

[B40] Santos M. A. D. (2011). Paidéia: relatório de gestão-2010. *Paidéia* 21 5–8. 10.1590/S0103-863X2011000100002

[B41] Santos M. A. D. (2012). Paidéia: relatório de gestão-2011. *Paidéia* 22 5–9. 10.1590/S0103-863X2012000100002

[B42] Santos M. A. D. (2013). Paidéia: relatório de gestão-2012. *Paidéia* 23 3–7. 10.1590/1982-43272354201302

[B43] Santos M. A. D. (2014). Paidéia: management report-2013. *Paidéia* 24 5–9. 10.1590/1982-43272457201402

[B44] Santos M. A. D. (2015). Paidéia: management report-2014. *Paidéia* 25 3–8. 10.1590/1982-43272560201502

[B45] Santos M. A. D. (2016). Paidéia: management report-2015. *Paidéia* 26 1–6. 10.1590/1982-43272663201601

[B46] Santos M. A. D. (2017). Paidéia: management report-2016. *Paidéia* 27 1–6. 10.1590/1982-43272766201701

[B47] Santos M. A. D. de Oliveira V. H. (2009). Paideia: relatório de gestão-2008. *Paidéia* 19 3–6. 10.1590/S0103-863X2009000100002

[B48] Santos M. A. D. Melo-Silva L. L. Risk E. N. (2008). Paidéia: cadernos de psicologia e educação: relatório de gestão 2007. *Paidéia* 18 13–20. 10.1590/S0103-863X2008000100002

[B49] SCImago (2017). *SJR - SCImago Journal & Country Rank*. Available at: https://www.scimagojr.com [accessed May 7 2017].

[B50] Smith M. J. Weinberger C. Bruna E. M. Allesina S. (2014). The scientific impact of nations: journal placement and citation performance. *PLoS One* 9:e109195. 10.1371/journal.pone.0109195 25296039 PMC4189927

[B51] Tijssen R. J. Mouton J. Van Leeuwen T. N. Boshoff N. (2006). How relevant are local scholarly journals in global science? A case study of South Africa. *Res. Eval.* 15 163–174. 10.3152/147154406781775904

[B52] Vasconcelos S. M. R. Sorenson M. M. Leta J. (2007). Scientist-friendly policies for non-native English-speaking authors: timely and welcome. *Braz. J. Med. Biol.Res.* 40 743–747. 10.1590/S0100-879X2007000600001 17581671

[B53] Wilson D. Purushothaman R. (2003). *Dreaming with BRICs: The path to 2050*, Vol. 99 New York, NY: Goldman, Sachs & Company

